# Effect of Diflunisal in Patients with Transthyretin Cardiomyopathy: A Pilot Study

**DOI:** 10.3390/jcm13175032

**Published:** 2024-08-25

**Authors:** Andrea Camblor Blasco, Ana Devesa, Luis Nieto Roca, Sandra Gómez-Talavera, Jairo Lumpuy-Castillo, Ana María Pello Lázaro, Lucía Llanos Jiménez, Javier Sánchez González, Óscar Lorenzo, Jose Tuñón, Borja Ibáñez, Álvaro Aceña

**Affiliations:** 1 Department of Cardiology, IIS-Fundación Jiménez Díaz University Hospital-Quiron Salud, 28040 Madrid, Spain; acamblor@hotmail.com (A.C.B.);; 2 Centro Nacional de Investigaciones Cardiovasculares Carlos III (CNIC), 28029 Madrid, Spain; 3 Mount Sinai Fuster Heart Hospital, New York, NY 10029, USA; 4 Centro de Investigación Biomédica en Red de Enfermedades Cardiovasculares (CIBERCV), 28029 Madrid, Spain; 5 Laboratory of Diabetes and Vascular Pathology, IIS-Fundación Jiménez Díaz, Universidad Autónoma, 28040 Madrid, Spain; 6 Biomedical Research Network on Diabetes and Associated Metabolic Disorders (CIBERDEM), Carlos III National Health Institute, 28029 Madrid, Spain; 7 Clinical Research Unit, Fundación Jiménez Díaz University Hospital, FJD Health Research Institute, Universidad Autónoma de Madrid (IIS-FJD, UAM), 28049 Madrid, Spain; 8 Faculty of Medicine, Universidad Autónoma de Madrid, 28049 Madrid, Spain

**Keywords:** heart failure, amyloid, transthyretin cardiomyopathy, diflunisal, stabilization

## Abstract

**Background:** ATTR-CM is becoming more prevalent, and disease-modifying therapy has been investigated in recent years with promising results. Diflunisal has shown TTR-stabilizing properties assessed by biomarkers and echocardiography, but there are no trials addressing the evolution of morphological changes with CMR. **Methods and Results:** AMILCA-DIFLU is an exploratory pilot study prospective, single-center, non-randomized, open-label clinical trial. Patients diagnosed with ATTR-CM underwent clinical, functional, biochemical and imaging assessment before and one year after diflunisal therapy initiation. Of the twelve ATTR-CM patients included, only nine patients completed treatment and study protocol in 12 months. To increase the sample size, we included seven real-world patients with one year of diflunisal treatment. Among the group of patients who completed treatment, diflunisal therapy did not show improvement in cardiac disease status as assessed by many cardiac and inflammatory biomarkers, 6MWT and CMR parameters after one year of treatment. However, a non-significant trend towards stabilization of CMR parameters such as LVEF, ECV and T2 at one year was found. When comparing the group of patients who completed diflunisal therapy and those who did not, a significant decrease in the distance performed in the 6MWT was found in the group of patients who completed treatment at one year (−14 ± 81.8 vs. −173 ± 122.2; *p* = 0.032). Diflunisal was overall well tolerated, showing only a statistically significant worsening in renal function in the group of diflunisal-treatment patients with no clinical relevance or need for treatment discontinuation. **Conclusions:** In patients with ATTR-CM, treatment with diflunisal was overall well tolerated and tended to stabilize or slow down amyloid cardiac disease progression assessed by CMR parameters, cardiac and inflammatory biomarkers and functional capacity.

## 1. Introduction

Cardiac amyloidosis is an infiltrative disease caused by the extracellular deposition of amyloid fibers in the myocardium, which leads to progressive heart failure (HF). When these deposited fibers are the result of misfolded transthyretin (TTR) protein, this condition is known as transthyretin cardiomyopathy (ATTR-CM). TTR is a liver-derived protein that transports thyroxine and retinol-binding protein. The accumulation of amyloid fibrils occurs when the tetrameric form disintegrates into monomers or oligomers due to pathogenic mutations in the transthyretin gene (ATTRm) or deposition of wild-type transthyretin protein (ATTRwt) [1].

This disease is becoming more prevalent due to diagnostic advances, with an estimated prevalence of 13% among patients with HF with preserved ejection fraction (HFpEF) and left ventricular (LV) hypertrophy, and in about 4–16% of cases of aortic stenosis in patients > 65 years, especially in those undergoing transcatheter aortic valve replacement according to previous reports [2,3]. Since HFpEF is a very prevalent disease, especially in elderly population [4], ATTR-CM management has a major impact on our health system.

To date, there is no curative therapy for ATTR-CM, and, until recently, treatment was limited to supportive HF therapy. However, disease-modifying therapies have been investigated in recent years with promising results and, at this time, some have been approved for ATTR-CM treatment. In 2018, the ATTR-ACT showed that tafamidis was associated with a reduction in all-cause mortality and hospitalizations for HF [5].

Also, diflunisal, a nonsteroidal anti-inflammatory drug (NSAID), has been demonstrated to bind and stabilize typical familial TTR variants, preventing acid-induced fibril formation in vitro [6], a mechanism similar to tafamidis, avoiding tetramer dissociation, and therefore amyloid fibrils accumulation. Studies in the last 10 years have reported that treatment with diflunisal was associated with efficacy in patients with ATTR polyneuropathy [7]. Although there are no controlled trials in the subset of groups with ATTR-CM, retrospective studies have suggested efficacy and stability of cardiac involvement according to echocardiographic and biomarker parameters [8,9,10] with overall good safety profile and tolerance of the drug in this population [11,12] and there is only one prospective study [12,13].

In addition, the preliminary results of the ATTRibute-CM Trial were recently presented. In this randomized control trial, acoramidis has been associated with a significant reduction in the incidence of the composite primary endpoint consisting of all-cause mortality, cumulative frequency of cardiovascular hospitalizations and change from baseline in NT-proBNP and 6MWT at 30 months as compared to placebo [14]. On the other hand, other novel treatments have mechanisms of action that are different from TTR-stabilizers, such as the TTR gene “silencers”, the TTR-specific small interfering RNAs (siRNAs) patisiran and vutrisiran and the antisense oligonucleotide (ASO) inotersen.

A recently published consensus regarding follow-up in these patients [15,16] has suggested a combined follow-up with three domains: (1) clinical and functional status, (2) laboratory biomarkers and (3) imaging and electrocardiographic parameters to assess disease progression. Cardiac magnetic resonance (CMR), especially T1 mapping, and ECV have been considered useful and objective parameters to address disease progression and efficacy of specific therapies [17]. Advanced echocardiographic evaluation using atrial and ventricular strain and myocardial work is gaining increasing interest [18,19]. In recently published trials, both patisiran and tafamidis have shown to slowdown cardiac disease progression assessed by CMR [20,21,22]. In this recent controlled trial [15], the authors even described a possible amyloid regression according to ECV reduction after one year under treatment with patisiran. These drugs are expensive and not always available for all patients; thus, a more cost-effective drug such as diflunisal could be useful in the treatment of ATTR-CM [23,24,25]. To date, no trials have addressed the evolution of morphological changes with CMR in patients under diflunisal therapy and ATTR-CM.

Therefore, the aim of our study was to describe the functional and morphological evolution of ATTR-CM patients assessed by several biomarkers, the 6MWT and CMR before and after one year of treatment with diflunisal.

## 2. Materials and Methods

### 2.1. Trial Oversight

AMILCA-DIFLU is an exploratory pilot study prospective, single-center, non-randomized, open-label clinical trial. The study took place in Hospital Fundación Jiménez Díaz and Centro Nacional de Investigaciones Cardiovasculares (CNIC) in Madrid, Spain from June 2021 to June 2023.

The study was approved by the institutional ethics committee (EUDRACT number 2019-002873-80) and Spanish Medicines Agency, and prospectively registered in EU Clinical Trials Register. All patients provided written informed consent. An external data- and safety-monitoring board was responsible for monitoring patient safety and conducted reviews of cumulative trial data.

To increase the sample size, we included all real-world patients who completed one year of treatment with diflunisal from February 2022 to June 2024. These patients were not initially part of the study due to having pacemakers (MRI scans) or because they began treatment after the clinical trial had already closed. During this period, in our hospital, all patients diagnosed with ATTR-CM were prescribed a stabilizing drug for the treatment of ATTR-CM (diflunisal or tafamidis since November 2023, when its use was approved in Spain). This study was approved by the institutional ethics committee (PIC 54/2017_FJD).

The study was conducted in accordance with the provisions of the Declaration of Helsinki and the guidelines of the International Conference on Harmonization Good Clinical Practice

### 2.2. Patients

All adult patients (>18 years of age) diagnosed with ATTR-CM (ATTRwt or ATTRm) in Hospital Fundación Jiménez Díaz were screened for inclusion.

Diagnosis was performed in accordance with to the latest ESC position statement on the diagnosis and management of cardiac amyloidosis criteria [15]. The diagnosis of ATTR-CM was established in all patients through advanced cardiovascular imaging techniques, including technetium pyrophosphate nuclear scintigraphy with confirmatory genetic testing. Grade 2 or 3 uptake on technetium-99m-pyrophosphate (TcPYP) scintigraphy, in the absence of a monoclonal gammopathy (normal serum free light chain ratio, no M protein on immunofixation), was considered diagnostic of ATTR-CM without the need for further invasive testing [15].

Patients were excluded if any of the following criteria was met: allergy to diflunisal or NSAIDs; previous gastrointestinal bleeding due to NSAIDs; life expectancy due to other comorbidities lower than one year; an estimated glomerular filtration rate (eGFR) lower than 30 mL per minute per 1.73 m^2^ of body surface area; liver transaminase levels exceeding two times the upper limit of the normal range; platelet count lower than 100,000 per microliter of blood; or being under treatment with two antiplatelet drugs in addition to anticoagulation therapy, previous cardiac or hepatic transplant, New York Heart Association (NYHA) class IV HF or pregnancy. In addition, patients with pacemakers or implantable cardioverter defibrillators and patients with any contraindication of CMR testing were also excluded from the initial study but were included in the real-world study.

### 2.3. Trial Design

All patients received diflunisal at a standard oral dose of 250 mg twice daily (bid). Patients who developed renal impairment were treated with lower doses (125 mg bid) at the discretion of study physicians.

All patients were prospectively followed for a year. The follow-up program consisted of clinical and functional assessments as well as biochemical and imaging testing one to seven days before initiation of diflunisal therapy. All tests were performed again after 12 months of treatment with diflunisal. In addition, clinical and routine laboratory follow-up was performed every three months while on diflunisal treatment, in accordance with safety protocols.

Concomitant use of other ATTR-specific treatments was permitted, although during the period this trial was conducted, neither tafamidis nor other specific ATTR-CM therapies had yet been approved in Spain for treatment of cardiac involvement of this disease.

#### Basal and 12-Month Protocol Studies

Data were collected from electronic health records at initiation visit and included baseline epidemiological and clinical characteristics, electrocardiographic and echocardiographic findings.

Physical examination, 12-lead-electrocardiogram and complete blood tests were performed in each study visit. In addition, before initiation of treatment and at one year of treatment, blood tests included baseline levels of both cardiovascular and inflammatory biomarkers: N-terminal pro-brain natriuretic peptide (NT-proBNP), N-terminal pro–atrial natriuretic peptide (NT-ProANP), high sensitivity Troponin I (hsTnI), creatine kinase-myocardial band (CK-MB), high sensitivity C-reactive protein (hsCRP), galectin 3 (GAL-3), interleukin 6 (IL-6), T cell immunoglobulin and mucin domain 1 (TIM1), matrix metallopeptidase-2 and -9 (MMP-2 and MMP-9), tissue inhibitors of metalloproteinases-1 and -2 (TIMP-1 and TIMP-2), retinol binding protein 4 (RBP4) and transthyretin (TTR, also called prealbumin). Certain biomarkers related to mineral metabolism, including parathyroid hormone (PTH), klotho, calcidiol (25(OH)D) and fibroblast growth factor 23 (FGF-23), were also assessed.

Standard transthoracic echocardiography was performed by a physician with expertise in cardiac imaging using a commercially available system (EPIQ 7C, Philips Medical Systems, Andover, MA, USA). CMR examinations were conducted at the CNIC between June 2021 and June 2023 using a Philips 3-T Elition X whole-body scanner (Philips Healthcare, Best, the Netherlands) equipped with a 28-element phased-array torso-cardiac coil. Echocardiography and CMR data were stored digitally, and studies were later analyzed at the CNIC Core Imaging Laboratory. T1 and T2 maps were automatically generated on the acquisition scanner. T1 and T2 values were extracted for basal, mid and apical segments, and average values were reported for each participant. Extracellular volume (ECV) was estimated with native T1 values and 15-min postcontrast T1 values corrected by hematocrit.

Functional assessment with a 6MWT was performed in all patients. Detailed information about laboratory, imaging and functional assessments described in the (Appendix A).

For real-world patients, all clinical data and echocardiographic parameters were collected prior to the start of diflunisal and after one year of treatment. Blood tests including hemoglobin, renal function and natriuretic peptides were also conducted.

Those patients who abandoned the treatment were considered non-completers and all tests were performed.

### 2.4. Statistical Analysis

All quantitative variables were examined for normal distribution (Shapiro–Wilk test). Quantitative data following a normal distribution are presented as mean ± standard deviation, those not normally distributed are displayed as median (interquartile range), and qualitative variables are presented as percentages. The differences in various parameters within the same subject were calculated using the paired t-test for those with normal distribution and the Wilcoxon signed-rank test for non-parametric ones. Differences in baseline data of patients with treatment as compared to those without treatment (non-completers) were assessed using X^2^ or Fisher’s exact test for qualitative data. For quantitative variables, a Student’s *t*-test was performed for those following a normal distribution, and the Mann–Whitney test was used for those not normally distributed.

The level of significance for all hypothesis tests was set at *p*-value < 0.05. All data were analyzed using SPSS version 20.0 software (IBM, Armonk, NY, USA) for Windows (Microsoft, Redmond, WA, USA).

## 3. Results

Twelve patients were included in the trial during the study period of June 2021 to June 2023. Nine patients completed treatment and study protocol for 12 months. Three patients abandoned diflunisal treatment prematurely and continued routine follow-up; there were no differences in any baseline characteristics between those who completed the study and those who did not. Among them, one patient abandoned the study voluntarily, after five days of treatment initiation other one after 10 days. The third patient who abandoned the study did so 158 days after treatment initiation after suffering a non-treatment-related, serious, non-lethal adverse event. Seven real-world patients, who completed one year of treatment with diflunisal, were also included. The group of patients who did not complete treatment were used for within-cohort comparisons with the treatment group, as it was considered that these three patients did not receive diflunisal therapy during most of the study protocol, and therefore followed the natural history of the disease.

### 3.1. Baseline Characteristics

Clinical baseline characteristics are shown in Table 1. Genetic testing was performed in all patients, with only two of them testing positive (hereditary ATTR-CM) and the rest negative (wild-type ATTR-CM). The two mutations found were Val50Met and Val142Ile. The median eGFR at baseline was 60.6 ± 14.6 mL/min/1.73 m^2^. Eight patients had a history of atrial fibrillation (50%), while seven of them were anticoagulated.

At baseline, 31.3% of the patients were under treatment with betablockers, 46.2% with angiotensin-converting enzyme inhibitors (ACEi) or angiotensin receptor blockers (ARB) and 31.3% with mineraloid receptor antagonists (MRA). Only one of the participants in our trial was receiving concomitant ATTR-specific treatment, patisiran 0.3 mg/kg intravenous infusion every three weeks due to evidence of neurological involvement and positive genetic testing (Val50Met). The other patient who tested positive (Val142Ile) had no evidence of neurological involvement at the time of trial participation, and therefore was not receiving any other ATTR-specific therapy during the study period.

Regarding serum biomarkers, the median NT-proBNP levels at baseline were 1815 (1132.5–3642.5) pg/L and hsTnI 154 ± 114.9 ng/mL.

Echocardiography showed that the left ventricular ejection fraction (LVEF) by Simpson biplane was 50 (46.1–58.5) % and GLSLV was −10.5 (−15.3 to −8.3) %. The median LVEF assessed by CMR was 57 (48–64) %, the median native T1 levels 1445.4 (1382–1467.1) ms, and the median ECV was 38 (33.1–49.3) %.

As for the assessment of functional capacity, all patients performed the 6MWT in accordance with study protocol, with a median baseline result of 260 (200–304) m.

Baseline characteristics of the group of patients who did not complete treatment with diflunisal at one year were similar to those described in the group of treatment patients and are displayed in the Supplementary Material (Table S1).

### 3.2. Diflunisal Tolerance

Overall, diflunisal was well tolerated. None of the patients withdrew diflunisal treatment due to adverse events related to the drug. All patients were treated with concomitant proton pump inhibitors, and none complained of gastrointestinal intolerance. No increase in the rates of bleeding were observed. Hemoglobin count remained stable in all patients. There was a significant drop in renal function measured as eGFR in the group of diflunisal treatment patients, as shown in Table 2 (60.6 ± 14.6 vs. 52.5.6 ± 14.7, *p* = 0.036). However, this renal worsening was not clinically relevant, and treatment withdrawal was not considered necessary for any of the patients. The diflunisal dose was decreased by half (125 mg twice daily) in two of the nine patients, with no further significant renal worsening.

During follow-up, one patient was hospitalized due to paroxysmal AF, two patients were admitted for HF, one patient required a pacemaker implantation, one patient was hospitalized for a respiratory infection, and one patient experienced central retinal artery occlusion.

### 3.3. Changes in Biomarkers

As shown in Table 2, there was no significant variability across basal and one-year determinations of NT-proBNP or hsTnI values.

TTR concentration levels significantly increased after one year under treatment with diflunisal (*p* = 0.008), as did RBP4 levels (*p* = 0.020).

### 3.4. Changes in Transthoracic Echocardiogram Parameters

As represented in Table 3, LVEF, right ventricular function (TAPSE), left ventricular filling parameters (E/E’) and vein cava dimenssion remained stable after one year of diflunisal therapy. At baseline 18.8% of patients had mitral regurgitation ≥ grade II and 18,8% of the patients had tricuspid regurgitation ≥ grade II and 6.3% and 12.5%, respectively, after one year of treatment.

### 3.5. Changes in Cardiac Magnetic Resonance Parameters

**Figure 1:**
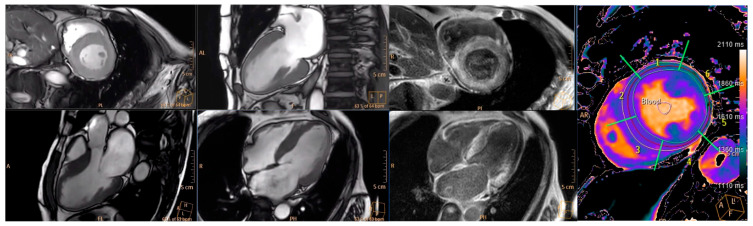
Example of cardiac magnetic resonance images showing typical pattern of ATTR-CM in one of the patients before diflunisal treatment initiation. Cine frames, contrast enhanced images and cardiac T1 mapping sequences are displayed on the left, medium and right of the figure, respectively.

As represented in Table 3, post-contrast T1 mapping, ECV (as a result of the non-significant reduction of post-contrast T1), T2 mapping, LVEF and LVMi remained stable after one year of diflunisal therapy. There was a significant slight increase in native T1 after one year of treatment (1424 ± 51.9 vs. 1446 ± 54; *p* = 0.004). Examples of CMR sequences acquired in one of the patients are displayed in Figure 1.

### 3.6. Functional Assessment

**Figure 2:**
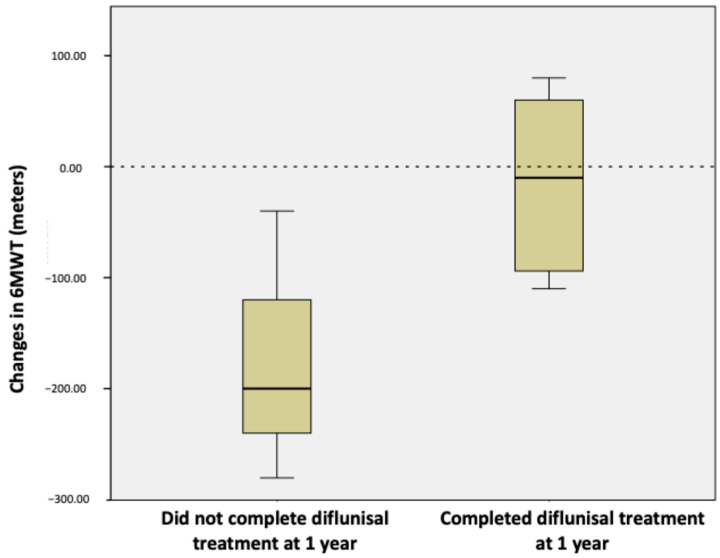
Graph illustrating the differences in performance of 6MWT from baseline to one-year follow-up between the group of patients who completed one-year treatment with diflunisal (n = 9) and the group who did not complete treatment with diflunisal (*n* = 3); *p*-value < 0.05 is considered significant.

There was a trend towards stability with a slight decrease in the distance in meters in the 6MWT after one year of follow-up (238 ± 66.7 vs. 223 ± 64.1 *p* = 0.626) (Table 3). The decrease in distance in performance of the 6MWT was significantly lower in the group of patients who completed treatment with diflunisal at one year compared to those who did not (−14 ± 81.8 vs. −173 ± 122.2; *p* = 0.032) (Figure 2).

## 4. Discussion

First, we found that diflunisal therapy in patients with ATTR-CM did not improve cardiac disease status as assessed by many cardiac and inflammatory biomarkers, 6MWT and CMR parameters after one year of treatment. This could be explained since diflunisal’s mechanism is to stabilize TTR protein by trying to reduce new amyloid deposition and further worsening, like other approved treatments, such as tafamidis, patisiran or inotersen, with no ability to reduce the amyloid burden present before treatment initiation. Taking into account diflunisal’s mechanism of action, in our study, we found a trend towards stabilization at one year of CMR parameters, such as LVEF, post contrast T1, ECV (as a result of the non-significant reduction of post-contrast T1) and T2, with only a slight significant increase in native T1 values.

To date, no trials have assessed the progression of ATTR-CM under diflunisal treatment with cardiac magnetic resonance parameters such as ECV. However, several recent trials have evaluated the effect of other TTR stabilizer (tafamidis) and MRA silencers (patisiran) with CMR parameters. In a recent trial, it was found that tafamidis 61 mg daily significantly delayed the progression of interstitial ECV expansion and LV mass, as well as halting deterioration of LV function in ATTR-CM patients [21]. Additionally, a study tried to analyze the benefit of tafamidis at one year in patients with ATTR-CM wild type based on serial CMR imaging and it found similar results and possible slowdown in cardiac disease progression measured by LVEF, LVMi, LVWT, native T1 and ECV compared to clear worsening in the group of treatment-naïve patients [22]. Moreover, patisiran has also recently shown cardiac amyloid regression in a study comparing patients under treatment with the medicine at one year retrospectively with matched patients who did not receive any disease-modifying therapy. Of note, 12 of the patients were concomitantly treated with diflunisal. The authors found that amyloid burden assessed by ECV diminished in 38% of the patients, was unchanged in 48% and increased in 19%. There were no significant differences in native T1 and T2 [20]. Recent consensus on ATTR-CM management [16] considers CMR parameters such as T1 mapping and ECV to be useful tools for assessment of disease progression. Also, elevated ECV and T2 mapping have previously been associated with worse prognoses [26].

In addition, in our study, we aimed to evaluate ATTR-CM progression assessing functional capacity through a 6MWT. We found a trend towards stabilization of the distance walked in the group of nine patients who completed treatment with diflunisal at one year (*p* = 0.626). Furthermore, after performing within-cohort comparisons between the group of patients who completed diflunisal treatment and those patients who did not, the worsening of performance of the 6MWT was significantly less in patients who were treated with diflunisal (*p* = 0.032). These results are similar to those found in the ATTR-ACT study in 2018 [5]. Moreover, the recent trial that evaluated the effect of patisiran on cardiac amyloid load through CMR parameters also found a significant improvement in 6MWT distances at 12 months in the ATTR-CM patients treated with patisiran [20].

Also, in our study, as previously described [12,27], we found a significant increase in plasma transthyretin (TTR) values after one year of diflunisal therapy compared with baseline. This is probably related to the fact that diflunisal stabilizes the structure of TTR tetramer and avoids its dissociation and potential deposition in cardiac tissue [9,28]. Accordingly, we also found higher levels of RBP4 after one year of treatment with diflunisal, which could also be explained by its interaction with the stabilized TTR.

In our study, we also measured cardiac failure-related biomarkers to assess disease progression. Changes in NT-proBNP levels were not significant after one year of diflunisal treatment. This is in line with previously published results and the expected absence of regression along with slowdown in natural disease progression [29,30]. We also measured several profibrotic and proinflammatory biomarkers that had previously been associated with an increase in amyloid disease [31]. We found an increased trend for IL-6, GAL-3 and TIMP-1. Most NSAID drugs can reduce inflammatory pathways such as cyclo-oxygenases (COX-1 and COX-2), which lead to decreases of prostacyclin and prostaglandin synthesis [32]. However, diflunisal can selectively interfere with some proinflammatory axes like the HMGB1/CXCL12/CXCR4 pathway but not others (i.e., the TLR4/MD-2 axis) [33]. Thus, diflunisal may not be able to reduce specific pro-inflammatory/fibrotic factors under amyloidosis, as previously observed [8].

The importance of stabilizing MRI parameters and various biomarkers lies in the indication that the amyloid burden in the patient’s heart is not increasing. Consequently, there is no progression of the disease, which is crucial as it prevents deterioration in exercise capacity, hospitalizations for heart failure, and mortality.

Overall, diflunisal was well tolerated, with no adverse clinical events related to treatment. In our study, we found a significant decline in renal function (*p* = 0.036) that led to a decrease in diflunisal doses by half (125 mg every 12 h) with amelioration or no further deterioration of renal function and no need for treatment discontinuation. This was not an unexpected finding since diflunisal is an NSAID that can cause renal toxicity, and, as previously reported [13], close renal surveillance of patients under treatment with diflunisal was performed.

One of the risks of NSAIDs is the potential for bleeding. This is important because patients with AF and TTR-CM need to be anticoagulated due to their high risk of embolism, but they also have a significant risk of bleeding. Therefore, it is crucial to monitor hemoglobin levels, inform patients of this risk, and adjust the diflunisal dose to 125 mg every 12 h if there is any doubt. Additionally, the option of left atrial appendage closure to reduce bleeding risk in these patients should be considered [34].

### Limitations

We acknowledge that a single-center, non-randomized study with a small sample size presents several limitations. First, the generalizability of the results is limited, as the findings may not be representative of the broader patient population. The non-randomized design introduces potential biases that could affect the validity of the conclusions.

Type I and type II errors could affect the statistical analysis. Therefore, we did a post hoc analysis of the statistical power for significantly changed variables, and we found that markers reached Prealbumin 0.61, RBP4 0.72, Native T1 0.74 and eGFR 0.88 of statistical power.

In addition, one of the major limitations of this study is the small sample size, with only 16 patients completing diflunisal at one year. Despite small size, this pilot study may serve as a preliminary step towards conducting larger, randomized, and multicentric studies to confirm these findings and provide more definitive evidence. ATTR-CM is considered a rare entity. Also, the inherent difficulties of the disease itself, including its the challenges in using NSAIDs and performing CMR with RTG in this subset of patients, further complicate the study and may influence the outcomes. Thus, studies with moderately large sample sizes are challenging. Indeed, non-randomized controlled trials evaluating diflunisal actions on cardiovascular risk biomarkers and echocardiographic parameters of patients with ATTR have been conducted in reduced cohorts. Additionally, it is worth noting that previous studies evaluating the efficacy of Tafamidis [21] and Patisiran [20] using CMR parameters had similarly small sample sizes, including 15 and 16 patients respectively. It is noteworthy that heterogeneous sources of data (protocol and real-life) can lead to biases.

Taking into account that (1) treatment with diflunisal in our study was tolerated well overall and a safe option in ATTR-CM patients as previously described [11,12]; (2) diflunisal treatment at one year tended to stabilize the natural progression of amyloid cardiac disease assessed by the 6MWT, CMR parameters and cardiac and proinflammatory biomarkers; and (3) diflunisal is an FDA-approved generic NSAID drug with widespread availability at a much lower cost than other specific therapies, with an estimated average annual diflunisal cost of USD 176 [24,35,36], we think that treatment with diflunisal should be taken into consideration in selected ATTR-CM patients, especially for those not eligible for other specific treatment options, as it may represent a well-tolerated, available and cost-effective alternative treatment. Our objective is to create a national registry of patients with ATTR-CM who are undergoing treatment with diflunisal and to attempt to conduct a multicenter randomized clinical trial to verify the benefit of this treatment. Moreover, a recent phase 1 randomized control trial has just been published addressing the safety of NI006, a recombinant human anti-ATTR antibody in patients with ATTR-CM and chronic HF. In this trial, this antibody seemed to reduce cardiac amyloid load assessed by cardiac tracer uptake on scintigraphy and extracellular volume on cardiac magnetic resonance imaging as well as cardiac biomarkers [37]. Further investigations are needed to address the possible combination of anti-ATTR antibody therapy as a first tackle to reduce amyloid load followed by chronic treatment with diflunisal.

## 5. Conclusions

In conclusion, in our series of patients with transthyretin amyloid cardiomyopathy, treatment with diflunisal 250 mg bid for one year was overall well tolerated and there seems to be a trend to stabilize or slow down amyloid cardiac disease progression assessed by CMR parameters, cardiac and inflammatory biomarkers and functional capacity. Further larger studies are required to validate the benefit of diflunisal therapy in ATTR-CM.

## Figures and Tables

**Table 1:** Baseline demographic and clinical characteristics.

VARIABLE	Patients Treated with Diflunisal *n* = 16
Age (years, IQR)	82.2 (78.5–87.0)
Male/Female	13/3
ATTRm/ATTRwt	2/14
**Comorbidities**	
Smoker (%)	37.5
Hypertension (%)	75.0
Diabetes mellitus (%)	12.5
Dyslipidemia (%)	75.0
Obesity (%)	18.8
Coronary artery disease (%)	11.1
Cerebrovascular disease (%)	6.3
Previous heart failure (%)	37.5
Atrial fibrillation (%)	50
Chronic kidney disease (%)	50.0
Carpal tunnel syndrome (%)	18.8
Lumbar canal stenosis (%)	18.8
Polyneuropathy (%)	12.5
**Previous Medical Treatment**	
Antiagregants (%)	6.3
Anticoagulants (%)	43.8
SLT2i (%)	31.3
ACEi (%)	18.8
ARB (%)	18.8
MRA (%)	31.3
Beta-blockers (%)	31.3
Antiarrhythmic drugs (%)	12.5
Digoxin (%)	0

Quantitative data are displayed as median (IQR = interquartile range), and qualitative variables are presented as percentages. ACEi = angiotensin-converting enzyme inhibitors; ARB = angiotensin receptor blockers; MRA = mineraloid receptor antagonists; SLT2i = sodium-glucose cotransporter 2 inhibitors.

**Table 2:** Baseline and one-year follow-up laboratory and biomarker determinations in patients who completed treatment with diflunisal.

VARIABLE	Visit 0	Visit 1	*p*
**Laboratory and Biomarker Determinations**			
Hb (g/dL± SD) (*n* = 16)	14.3 ± 1.7	14.0 ± 1.8	0.263
eGFR (mL/min/1.73 m^2^, ± SD) (*n* = 16)	60.6 ± 14.6	52.5 ± 14.7	**0.036**
NT-proBNP (pg/mL, IQR) (*n* = 16)	1815.0 (1132.5–3642.5)	2020.0 (836.25–3997.5)	0.272
HsTnI (pg/mL, ± SD) (*n* = 9)	154.6 ± 114.9	153.1 ± 137.8	0.971
IL-6 (pg/mL, IQR) (*n* = 9)	2.7 (2.4–36.2)	3.0 (1.3–6.3)	0.08
TIM-1 (pg/mL, ± SD) (*n* = 9)	199.6 ± 104.2	232.8 ± 131.6	0.359
Gal3 (pg/mL, ± SD) (*n* = 9)	7544.7 ± 2307.5	8945.4 ± 3926.3	0.303
hsCPR (mg/L, ± SD) (*n* = 9)	2.4 ± 2.8	2.3 ± 1.7	0.952
CK-MB (ng/mL, ± SD) (*n* = 9)	1.7 ± 0.7	2.6 ± 1.3	0.165
NT-ProANP (ng/mL, ± SD) (*n* = 9)	65.2 ± 12.6	64.5 ± 12.2	0.790
PTH (pg/mL, ± SD) (*n* = 9)	78.9 ± 32.0	90.0 ± 43.0	0.418
Klotho (pg/mL, ± SD) (*n* = 9)	535.4 ± 124.6	564.5 ± 169.4	0.669
FGF-23 (RU/mL, IQR) (*n* = 9)	257 (126.5–328.5)	149 (131–207)	0.499
VitD (ng/mL, ± SD) (*n* = 9)	33.1 ± 13.2	34.7 ± 9.6	0.776
RBP4 (µg/mL, ± SD) (*n* = 9)	28.0 ± 10.5	33.6 ± 13.0	**0.020**
TIMP-1 (ng/mL, ± SD) (*n* = 9)	183.5 ± 31.3	201.8 ± 33.1	0.069
TIMP-2 (ng/mL, ± SD) (*n* = 9)	116.9 ± 12.6	124.0 ± 22.9	0.371
MMP-2 (ng/mL, ± SD) (*n* = 9)	330.2 ± 47.0	339.1 ± 54.9	0.619
MMP-9 (ng/mL, IQR) (*n* = 9)	365 (228–571)	497.5 (227.8–966.0)	0.515
Prealbumin (mg/dL, IQR) (*n* = 9)	26.5 (19.6–28.7)	32.3 (27.3–33.6)	**0.008**

Quantitative data following a normal distribution are presented as mean ± standard deviation (SD), those not normally distributed are displayed as median (IQR = interquartile range), and qualitative variables are presented as percentages. CK-MB = creatine kinase-myocardial band; eGFR = estimated glomerular filtration rate; FGF-23 = fibroblast growth factor 23; GAL-3 = galectin 3; Hb = hemoglobin; hsCRP = high sensitivity C-reactive protein; hsTnI = high-sensitivity troponin I; IL-6 = interleukin 6; MMP-2 = matrix metallopeptidase-2; MMP-9 = matrix metallopeptidase-9; NT-proANP = N-terminal pro–atrial natriuretic peptide; NT-proBNP = N-terminal pro-brain natriuretic peptide; PTH = parathyroid hormone; RBP4 = retinol binding protein 4; TIM-1 = T cell immunoglobulin and mucin domain 1; TIMP-1 = tissue inhibitors of metalloproteinases-1; TIMP-2 tissue inhibitors of metalloproteinases-2.

**Table 3:** Baseline and one-year follow-up imaging and functional parameters inpatients who completed treatment with diflunisal.

VARIABLE	Visit 0	Visit 1	*p*
**Transthoracic Echocardiogram**			
TAPSE (mm, IQR) (*n* = 16)	16.5 (15.3–21.0)	18.0 (13.5–27.5)	0.285
LVEF Simpson biplane (%, ± SD) (*n* = 16)	49.8 ± 11.0	51.9 ± 9.6	0.303
E/E’ (*n* = 16)	16.2 ± 4.6	16.3 ± 4.7	0.947
Vein cava diameters (mm) (*n* = 16)	16.0 (15.0–18.0)	15.5 (14.3–17.0)	0.752
GLS LV (%, ± SD) (*n* = 9)	−11.2 ± 3.9	−10.6 ± 5.3	0.728
GLS RV (%, ± SD) (*n* = 9)	−10.3 ± 4.5	−9.7 ± 3.5	0.523
GLS LA (%, ± SD) (*n* = 9)	9.1 ± 5.6	6.4 ± 6.8	0.181
**Cardiac Magnetic Resonance**			
LVEF (%, ± SD) (*n* = 9)	56 ± 7.2	59 ± 8.3	0.376
LVMi (g/m^2^, ± SD) (*n* = 9)	53.2 ± 32.3	71.3 ± 36.0	0.139
Native T1 (ms, ± SD) (*n* = 9)	1424.9 ± 51.9	1446.9 ± 54.8	**0.004**
Post-contrast T1 (ms, ± SD) (*n* = 9)	524.8 ± 68.1	499.7 ± 52.9	0.130
ECV (%, ± SD) (*n* = 9)	40.7 ± 8.3	42.7 ± 9.4	0.415
T2 (ms, IQR) (*n* = 9)	55.3 (52.7–58.7)	58.8 (54.6–60.2)	0.401
**6-min Walk Test (6MWT)**			
Distance (m, ± SD) (*n* = 9)	238.5 ± 66.7	223.8 ± 64.1	0.626

Quantitative data following a normal distribution are presented as mean ± standard deviation, those not normally distributed are displayed as median (interquartile range), and qualitative variables are presented as percentages. ECV = extracellular volume; GLS = global longitudinal strain; LA = left atrium; LVMi = left ventricular mass indexed; LV = left ventricle; LVEF = left ventricular ejection fraction; RV = right ventricle; TAPSE = tricuspid annular plane systolic excursion.

## Data Availability

The original contributions presented in the study are included in the article/Supplementary Material, further inquiries can be directed to the corresponding author.

## Supplementary Materials

The following supporting information can be downloaded at: https://www.mdpi.com/article/10.3390/jcm13175032/s1, Table S1: Baseline and one-year follow-up laboratory, imaging and functional parameters in patients who did not complete treatment with diflunisal.

## Appendix A

### Appendix A.1. Biomarker Determination

According to biomarker determination, plasma concentrations of human MCP1, hsCPR, TIM 1, IL-6 and galectin 3 were measured using the automated immunoassay system ELLA from Protein Simple (Bio-Techne, Minnesota, MN, USA), following the manufacturer’s instructions. The detection kits were SPCKA-PS-008754 (TIM1 and IL-6), SPCKB-PS001108 (MCP1), SPCKB-PS000200 (hsCRP) and SPCKB-PS000490 (galectin-3). Each plasma sample was run in triplicate, and the inter-plate coefficient of variation was less than 4% in all cases. The levels of plasma human NT-ProANP were obtained by using immunoassay using specific kits (ref: DANP00) from R&D Systems, and those of human creatine kinase–myocardial band (CK-Mb) were achieved by immunoassay using VITROS Immunodiagnostic products (ref: 1896836) at the Analytical Service of the Fundación Jiménez Díaz. The plasma concentration of human, hsCPR, TIM 1, IL-6, galectin 3, MMP-9 and TIMP-1 were measured using the automated immunoassay system ELLA from Protein Simple (Bio-Techne, Minneapolis, MN, USA), following the manufacturer’s instructions. The detection kits were SPCKB-PS000200 (hsCPR), SPCKA-PS-008754 (TIM1 and IL-6), SPCKB-PS000490 (galectin-3), SPCKC-PS-000661 (MMP-9) and SPCKC-PS-001442 (TIMP-1). Each plasma sample was run in triplicate, and the intra-assay coefficient of variation was less than 4% in all cases. Additionally, the enzyme-linked immunosorbent assays (ELISA) were utilized to quantify MMP-2 (MMP200, Bio-Techne, Minneapolis, MN, USA), TIMP-2 (DTM200, Bio-Techne, Minneapolis, MN, USA), RBP4 (DRB400, Bio-Techne, Minneapolis, MN, USA) and NT-ProANP (DANP00, Bio-Techne, Minneapolis, MN, USA). The absorbance was set at 450 nm with a wavelength correction at 570 nm using a plate reader (EnSpire® Multimode Reader, Perkin Elmer, Waltham, MA, USA). Also, FGF23 was measured by an enzyme-linked immunosorbent assay that recognizes epitopes within the carboxyl-terminal portion of FGF23 (Human FGF23, C-Term, Immutopics Inc, San Clemente, CA, USA), and klotho levels were measured by ELISA (human soluble alpha-klotho assay kit, Immuno-Biological Laboratories Co., Hokkaido, Japan). Intact PTH was analyzed by a second-generation automated chemiluminescent method (Elecsys 2010 platform, Roche Diagnostics, Mannheim, Germany). Finally, the plasma levels of CK-MB and prealbumin (TTR) were determined through immunoassay at the Clinical Biochemistry General Laboratory of Fundación Jiménez Díaz in Madrid, Spain. The respective detection kits employed were CK-MB (CK-MB reagent pack, ref: 1896836, VITROS Immunodiagnostic, Raritan, NJ, USA) and prealbumin (PREA, ref: 05950902190, ROCHE, Basel, Switzerland).

### Appendix A.2. Image Acquisition Transthoracic Echocardiography

Standard transthoracic echocardiography was performed by a physician with expertise in cardiac imaging using a commercially available system (EPIQ 7C, Philips Medical Systems, Andover, MA, USA). Echocardiography data were stored digitally. Ultrasound studies were later analyzed with QLab9 (Philips Healthcare, Bothel, WA, USA) at the Centro Nacional de Investigaciones Cardiovasculares (CNIC) Core Imaging Laboratory. Imaging quality was evaluated as optimal, suboptimal or noninterpretable, and inclusion of studies was determined by consensus. Ejection fraction (EF) was measured with the biplane Simpson method in apical four- and two-chamber views. LV mass and mass index (LVMi) were assessed through the Cube formula. Myocardial volume was calculated as the ratio of LV mass over 1.05 (myocardial density). Mitral inflow early (E) and late diastolic (A) velocities, the (E/A) ratio and deceleration time of E wave (DT) and the pulsed wave tissue doppler early diastolic mitral velocity (E′) of the medial and lateral annulus were measured. Right ventricular size was defined using basal end–diastolic diameter, function was defined by assessing the tricuspid annular plane systolic excursion (TAPSE) and S wave was obtained by pulse wave tissue doppler. Doppler tracings of the aortic and mitral valve were used to define end systole and end diastole. All reported measurements were averaged over three cardiac cycles. LV, RV and LA strain measurements were performed offline, as recommended. Digitally acquired clips were considered suitable for offline 2D speckle strain imaging analysis when at least three cardiac cycles were available, with high frame rates (70 to 100 frame/s) and without dropout of more than one LV segment. The endocardial border was traced at the end-diastolic frame in the apical view. End-diastole was defined by the QRS complex or by the frame just before mitral valve closure. The software tracked speckles along the endocardial and epicardial borders throughout the cardiac cycle, and the width of the region of interest was adjusted to fit the entire myocardium. All strain and strain-derived variables were measured in the apical four-chamber view.

### Appendix A.3. Cardiac Magnetic Resonance Protocol and Image Analysis

CMR examinations were conducted at the CNIC between June 2021 and June 2023, using a Philips 3-T Elition X whole-body scanner (Philips Healthcare, Best, the Netherlands) equipped with a 28-element phased-array torso-cardiac coil. Body weight and height were measured immediately before the CMR examination. The cardiac imaging protocol included a standard segmented cine steady-state free-precession (SSFP) sequence to provide high-quality images for the assessment of cardiac chamber dimensions and function. The imaging protocol also included a late gadolinium enhancement (LGE) study assessed 15 min after the administration of intravenous bolus of 0.10 mmol/Kg gadoteric acid contrast agent using inversion recovery spoiled TFE acquisition, as well as three short axis slices (basal, mid and apical) T2 gradient-spin-echo (T2-GraSE) mapping sequence and three short axis slices (basal, mid and apical) 5(3)3 modified look-locker inversion recovery (MOLLI) T1-mapping sequence for myocardial tissue characterization, which was applied prior to contrast agent administration and 15 minutes afterwards.

### Appendix A.4. CMR Analysis

Images were analyzed by experienced observers (A.C., A.D.) using a dedicated software program available at the CNIC imaging core lab (IntelliSpace Portal v12.1, Philips Haifa, Haifa, Israel).

Analysis of ventricular volumes and function as well as LV mass was made by manually contouring, tracing the endocardial and epicardial contours in end-diastole and end-systole on the short-axis cine images. Scar extension was defined both visually by experienced observers and quantitatively evaluated after manually tracing the endocardial and epicardial contours on LGE short-axis images. Abnormal areas were defined using a cutoff of mean remote myocardium intensity plus 2.5 standard deviations. T1 and T2 maps were automatically generated on the acquisition scanner. T1 and T2 values were extracted for basal, mid and apical segments, and average values were reported for each participant. Extracellular volume (ECV) was estimated with native T1 values and 15-minute postcontrast T1 values corrected by hematocrit.

### Appendix A.5. Functional Assessment. Six-Minute Walk Test (6MWT)

In a quiet 40-meter-long hall at the CNIC, the patients were instructed to walk as fast as possible, completing as many laps as possible between the distance markers over a period of six minutes. The walk test was supervised but not encouraged. Blood pressure and heart rate were measured at the start and at the end of the test. Patients were allowed to stop and rest if needed. The total distance walked was recorded.
