# Supplementary material for: Effect of Diflunisal in Patients with Transthyretin Cardiomyopathy: A Pilot Study
Source: J Clin Med. 2024 Aug 25;13(17):5032. doi: 10.3390/jcm13175032 (PMC11396251; doi:10.3390/jcm13175032)
Supplement: Supplementary file 1 [file jcm-13-05032-s001.zip › jcm-3145711-supplementary.pdf]

Table S1. Baseline and one-year follow-up laboratory, imaging and functional parameters in patients who did not complete treatment with diflunisal.

VARIABLE	Visit 0	Visit 1	P
**Laboratory and biomarker determinations**			
Hb (g/dL,± SD)	13.1±1.5	13.3±1.8	0.711
eGFR (mL/min/1.73m2, ± IQR)	74.9 (54.3–80.7)	68 (29–68)	0.600
NT-proBNP (pg/mL,± SD)	1295.3±682.6	2056±1551	0.105
HsTnI (pg/mL, IQR)	113.6 (41.8–135)	145.9 (93.6–145.9)	0.285
IL-6 (pg/mL, IQR)	5.8 (3.1–5.7)	6.4 (2.6–6.4)	1.000
TIM-1 (pg/mL, IQR)	172 (81.1–172)	263 (84–263)	0.109
Galectin 3 (pg/mL, IQR)	7475 (6568.8–8975.2)	10670 (8080–10670)	0.285
HsCPR (mg/L, IQR)	1.73 (0.8–2.4)	1.5 (0.6–1.5)	0.593
CK-MB (ng/mL, IQR)	3.6 (2.2–12.5)	2.7 (0.5–2.7)	0.593
NT-proANP (ng/mL, IQR)	73 (68.9–73)	59.7 (55.5–59.7)	0.285
PTH (pg/mL, IQR)	130 (35.5–130.5)	84.3 (32.2–84.3)	1.000
Klotho (pg/mL, IQR)	628 (525–628)	765 (534–765)	0.285
FGF-23 (RU/mL, IQR)	499.5 (208–499.5)	485.5 (185–485)	0.180
VitD (ng/mL, IQRl	35.7 (32–35.7)	36.1 (28.1–36.1)	0.285
RBP4 (µg/mL, IQR)	24.8 (18.3–24.8)	27 (20.7–27)	0.109
TIMP-1 (ng/mL, IQR)	153.7 (151.6-153.7)	238.3 (166.8-238.3)	0.109

TIMP-2 (ng/mL, IQR)	127.2 (120.1–127.2)	139.9 (96.1–139.9)	1.000
MMP-2 (ng/mL, IQR)	351.2 (299.1–351.2)	395.3 (304.9–395.3)	0.593
MMP-9 (ng/mL, IQR)	153.6 (77.1–153.6)	286.0 (247.6–286.0)	0.109
Prealbumin(mg/dL, IQR)	20.8 (19.2–21)	29 (27.5–29.0)	1.000
**Transthoracic echocardiogram**			
TAPSE (mm, IQR)	18 (16–18)	18 (17–18)	0.655
LVEF Simpson biplane (%, ± SD)	56.3±19.7	32.1±37.3	0.291
GLS LV (%, IQR)	-12 (-21 to -12)	-17 (-11.1 to -17.8)	0.180
GLS RV (%, IQR	-10.4 (-10.8 to -10.4)	-11.4 (-15.8 to -11.4)	0.655
GLS LA (%, ± IQR)	5.5 (1.3–5.5)	10.3 (10–10.3)	0.180
**Cardiac magnetic resonance**			
LVEF (%, ±SD)	59.5±6.6	59.3±12.2	0.945
LVMi (g/m2, ± IQR)	74 (50–109.6)	?	0.225
Native T1 (ms, ± SD)	1292.1±144.4	1665.5±371.2	0.492
Post-contrast T1 (ms, IQR)	565 (546.6–565.3)	549(549–549)	0.317
ECV (%, IQR)	38.6 (31.1–43.9)	38 (38–38)	0.655
T2 (ms, ± SD)	66.7±17.9	77.9±28.9	0.386
**Six-minute-walk test (6MWT)**			
Distance (m, ± SD)	300±20	126.7±130.1	0.133

Quantitative data following a normal distribution are presented as mean ± standard deviation, those not normally distributed are displayed as median (interquartile range) and qualitative variables are presented as percentages.

CK-MB = creatine kinase-myocardial band; ECV = extracellular volume; eGFR = estimated glomerular filtration rate; FGF-23 = fibroblast growth factor 23; GAL-3 = galectin 3; GLS = global longitudinal strain; Hb = hemoglobin; hsCRP = high-sensitivity C-reactive protein; hsTnI = high-sensitivity troponin I; IL-6 = interleukin 6;LA = left atrium; LVMi = left ventricular mass indexed; LV = left ventricle; LVEDD = left ventricular end diastolic diameter; LVEF = left ventricular ejection fraction; LVESD = left ventricular end systolic diameter; MMP2 = matrix metallopeptidase-2; MMP9 = matrix metallopeptidase-9; NT-proANP = N-terminal pro–atrial natriuretic peptide; NT-proBNP = N-terminal pro-brain natriuretic peptide; PTH = parathyroid hormone; RBP4 = retinol binding protein 4; RV = right ventricle; TAPSE = tricuspid annular plane systolic excursion; TIM1 = T cell immunoglobulin and mucin domain 1; TIMP-1 = tissue inhibitors of metalloproteinases-1; TIMP-2 tissue inhibitors of metalloproteinases-2.
